# Polycythemia Vera With High Serum Erythropoietin Level: A Case Report and Literature Review

**DOI:** 10.7759/cureus.69578

**Published:** 2024-09-17

**Authors:** Ahmed Alsayed, Abdalla Fadul, Muzamil Musa, Motaz M Almahmood, Khalid Albsheer, Amjad M Salman, ELMustafa Abdalla

**Affiliations:** 1 Internal Medicine Department, Hamad Medical Corporation, Doha, QAT

**Keywords:** hematocrit, jak-2 mutation, polycythemia vera, secondary polycythemia, serum erythropoietin

## Abstract

Polycythemia vera (PV) is a primary acquired marrow condition that causes erythrocytosis. It may present with erythromelalgia, pruritus, splenomegaly, and thrombotic events. Secondary causes of polycythemia should be ruled out before labeling a patient as having PV. Serum erythropoietin (EPO) level helps distinguish primary and secondary polycythemia, but it should be aided by further testing such as the JAK-2 gene mutation test. We present a case of a previously healthy 47-year-old female who came to the hospital with a headache and transient left-sided body weakness. She had no similar episodes in the past. Her initial workup showed a high hemoglobin and a high hematocrit level. A plan computed tomography (CT) scan of the head showed evidence of a thalamo-capsular infarct. The serum EPO level was elevated, and a bone marrow biopsy returned positive for JAK-2 mutation indicating the diagnosis of polycythemia vera despite the high EPO level. The World Health Organization (WHO) consensus criteria for diagnosing PV demand the presence of two major criteria and one minor criterion or the presence of the first major criterion and two minor criteria. Decreased EPO is considered a minor WHO criterion for PV diagnosis. A low EPO is also used to discriminate PV from secondary thrombocytosis, as it might be low, as expected, or elevated. Phlebotomy primarily treats PV with low risk with a target hematocrit of less than 45%. PV patients with high risk can benefit from low-dose aspirin. Anticoagulation may be added for patients with thromboembolism. Patients with polycythemia vera can present with a high or low serum EPO level. Further diagnostic tests are usually required to confirm the final diagnosis.

## Introduction

Polycythemia vera (PV) is a condition that affects bone marrow (BM) stem cells and is characterized by an increase in red blood cell mass that is frequently accompanied by fluctuating elevations in leucocyte and platelet counts. Patients with PV are at risk for thrombosis due to the hyperviscosity of the blood [1]. So far, only PV, as opposed to secondary erythrocytosis from diverse extramedullary pathological events, is a primary acquired marrow condition that causes erythrocytosis. Although reported PV prevalence varies by area, it is often more prevalent in older people. Sixty years is the average age at diagnosis [2]. High blood counts can cause a variety of symptoms, such as erythromelalgia, migraines, complicated headaches, and transient ischemic episodes. In rare instances, patients may also feel bone discomfort, nocturnal sweats, and itching [3]. The key diagnostic criteria set by the World Health Organization (WHO) are a high level of hemoglobin or hematocrit, abnormal results from a BM biopsy, and the presence of the genetic mutation Janus kinase 2, which is present in almost 98% of cases. A subnormal erythropoietin level is the sole minor factor that helps distinguish PV from frequent causes of secondary erythrocytosis, including testosterone usage, sleep apnea, and smoking [4]. Even before serum EPO level was included as a WHO diagnostic criterion, it was commonly believed that a raised EPO level suggested secondary erythrocytosis and excluded PV [5]. All this is illustrated in our case report.

## Case presentation

A previously healthy 47-year-old female presented complaining of severe frontal headache radiating to her neck, associated with dizziness and epigastric pain. She also reported transient left-sided body weakness for one day before this presentation, which ultimately resolved later. The systemic review was utterly unremarkable. Her initial vital signs were as follows: temperature 36.8 ºC, blood pressure 166/82 mmHg, pulse 76 bpm, oxygen saturation above 98% on ambient air, and a respiratory rate of 20 BR/min. Physical examination was completely unremarkable apart from the plethoric face. Initial laboratory test results are shown in Table 1.

**Table 1:** Initial laboratory test results WBC: white blood cell, RBC: red blood cell, MCV: mean corpuscular volume, ALP: alkaline phosphatase, ALT: alanine transaminase, AST: aspartate aminotransferase

Detail	Value w/units	Flags	Normal range
WBC	25.4 x10^3/µL	High	4.0-10.0
RBC	5.8 x10^6/µL	High	3.8-4.8
Hemoglobin	17.1 g/dL	High	12.0-15.0
Hematocrit	52.2%	High	36.0-46.0
MCV	90.2 fL	Normal	83.0-101.0
Platelets	547 x10^3/µL	High	150-400
Basophil	0.52 x10^3/µL	High	0.02-0.10
Urea	5.4 mmol/L	Normal	2.5-7.8
Creatinine	100 µmol/L	High	44-80
Sodium	140 mmol/L	Normal	133-146
Potassium	4.7 mmol/L	Normal	3.5-5.3
Bicarbonate	22 mmol/L	Normal	22-29
ALP	173 U/L	High	35-104
ALT	20 U/L	Normal	0-33
AST	29 U/L	Normal	0-32

**Figure 1:**
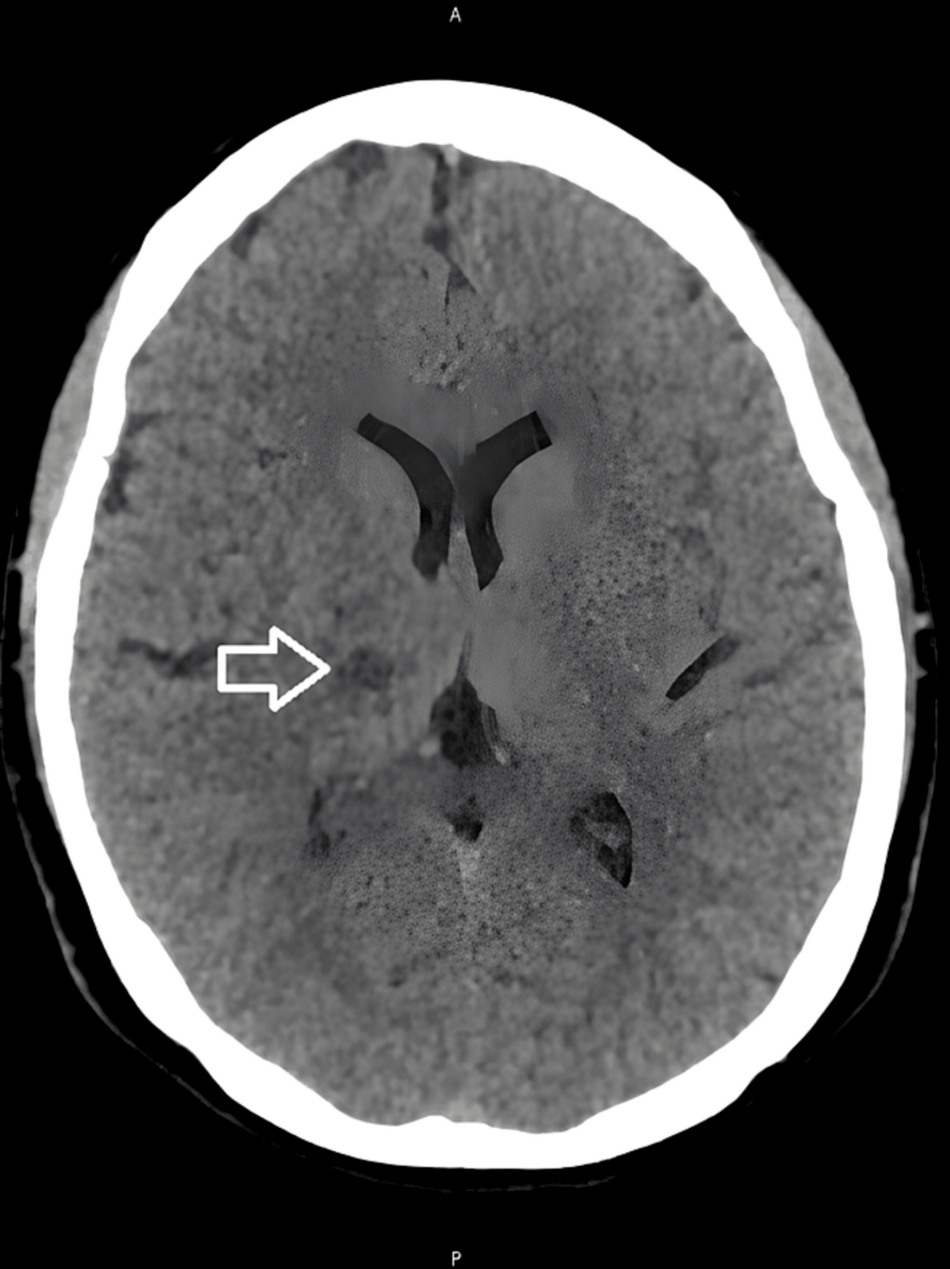
A plain CT head showing a focal hypodensity in the right capsulothalamic region suggestive of a recent infarct.

**Figure 2:**
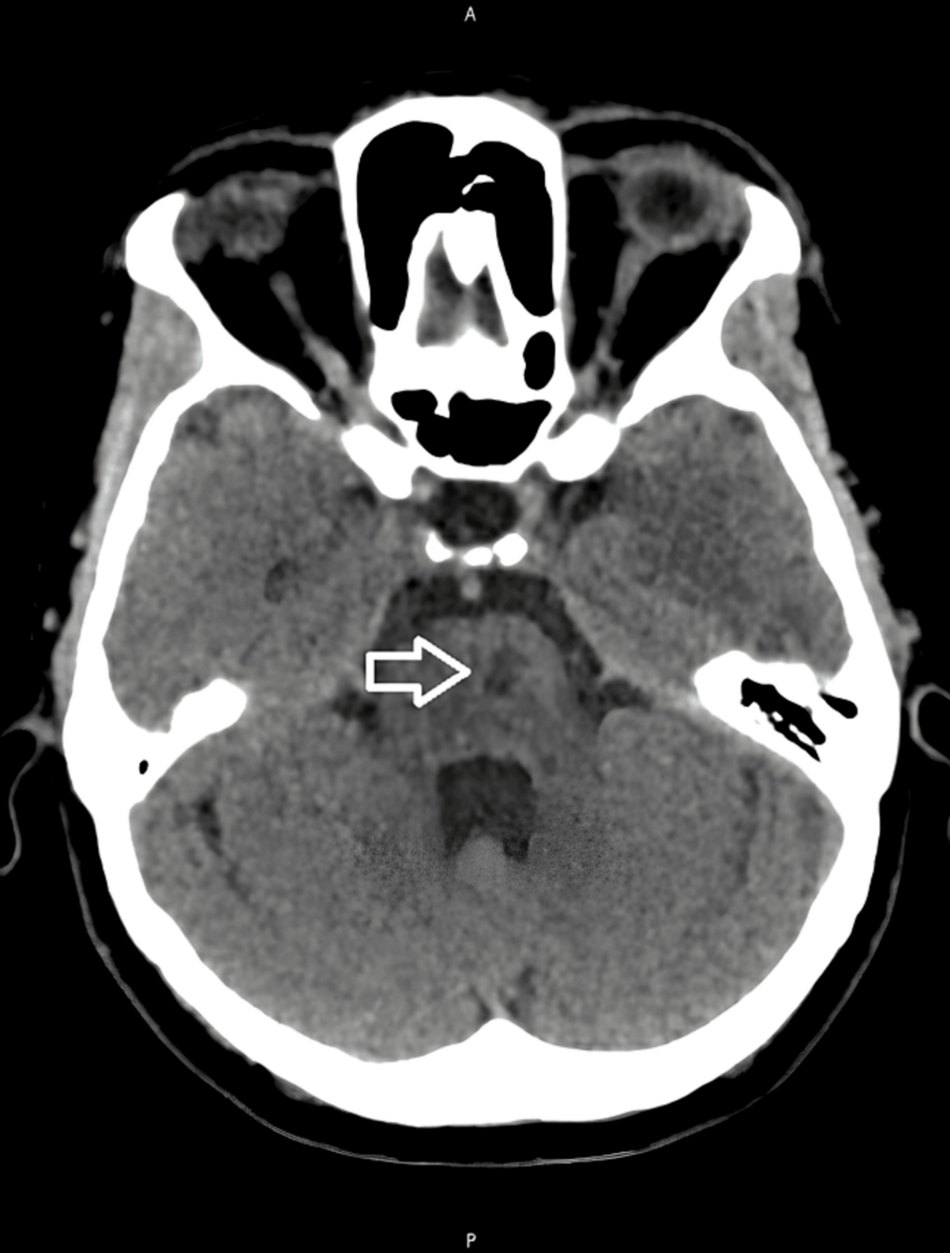
A plain CT head showing a left paramedian pons hypodensity suggestive of an infarct of indeterminant age.

**Figure 3:**
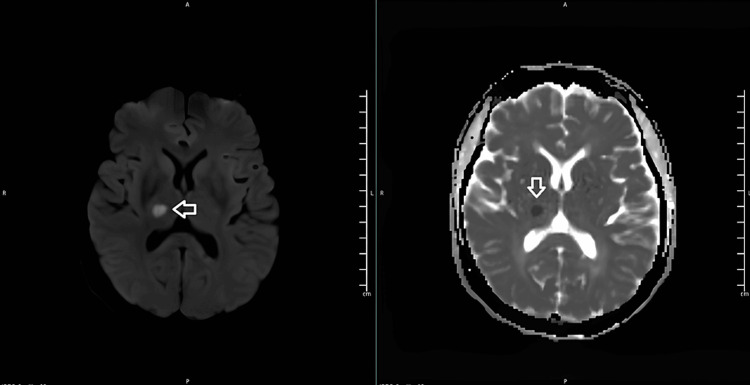
An MRI head DWI (left) and ADC (right) images showing a right thalamus acute to early subacute infarct. DWI - Diffusion-weighted imaging, ADC - apparent diffusion coefficient

**Figure 4:**
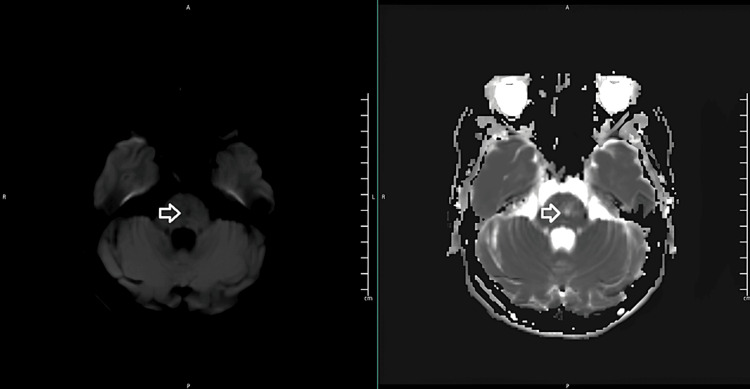
An MRI head DWI (left) and ADC (right) images showing a left paramedian pons lacunar infarct. DWI - Diffusion-weighted imaging, ADC - apparent diffusion coefficient

A head CT scan revealed a focal hypodensity in the right capsulothalamic area suggesting a recent infarct as well as a hypodensity in the left paramedian pons suggesting an infarct of unknown age (Figures 1, 2). A right thalamus acute to early subacute infarct was discovered during a subsequent head MRI (Figures 3, 4).

Her electrocardiogram was completely unremarkable. Subsequently, her peripheral smear showed erythrocytosis, neutrophils leukocytosis, eosinophilia, basophilia, and moderate thrombocytosis with few giant platelets. Based on the above, the EPO level sent was 138.01 mIU/mL (normal range 2.59-18.50 mIU/mL). An ultrasound abdomen showed normal spleen and liver size with no apparent renal mass or intra-abdominal abnormalities. The hematology team recommended a whole-body CT scan to rule out malignancy; however, it was not done due to acute kidney injury. Subsequently, a BM biopsy showed myeloproliferative neoplasm with features of PV, which were confirmed later by a positive test for JAK-2 mutation. The patient was treated as a case of PV and discharged home with regular hematology follow-up. Her repeated EPO level in the follow-up clinic after treatment was 4.78 mIU/mL.

## Discussion

The abnormal proliferation of PV is sustained by oncogenic mutations in the JAK-STAT path (signal transducers and activators of transcription). The most common mutation is the V617F mutation within exon 14 which represents 95% of the cases. Other mutations include exon 12 of the JAK2 gene which represents 4% of the cases [6]. A new set of diagnostic criteria for PV was proposed by the WHO in 2001. These criteria consider the knowledge that has been gained about the clinical and genetic characteristics of the illness [7]. A BM biopsy with hypercellularity, proliferative erythroid, granulocytic, and megakaryocytic lineages; an endogenous erythroid colony formation in vitro; and a low serum EPO level are the minor diagnostic criteria [6,8,9]. A low EPO is also used to discriminate PV from secondary thrombocytosis because it is usually seen in PV. However, a high EPO level can happen in PV sometimes leading to more confusion in the diagnostic process [9]. Exploring a secondary cause of polycythemia is recommended whenever the EPO level is high [10].

Thrombotic conditions including deep vein thrombosis, coronary artery clots, and hepatic blood clots leading to Budd Chiari syndrome, stroke, and splenic infarction are among PV consequences. Blood clotting activation in PV is caused by a number of abnormalities in platelets, RBCs, and WBCs as well as malfunctioning endothelial cells [8]. PV care should incorporate risk stratification based on age (60 versus >60 years) and history of a prior thrombosis. Low-risk patients are those who are under 60 years old and have no history of thrombosis, while high-risk patients are those who fall into any other category. Low-dose aspirin can also be used for high-risk PV, with phlebotomy being the main treatment for low-risk PV to keep the hematocrit below 45%. Additionally, typical management includes cytoreductive therapy with hydroxyurea, interferon, or busulfan. Anticoagulation should be administered at the correct doses and intensities to patients who have PV and thrombotic events. The best time to start anticoagulation in a patient with PV and the first episode of venous thromboembolism, however, has not been investigated in any randomized investigations [11]. As far as we are aware, there are just a few case reports in the literature that describe primary polycythemia cases with elevated blood EPO levels [12,13]. Budd Chiari syndrome, which did not exist in our instance, was linked to both cases.

## Conclusions

Serum EPO level is usually the next step in evaluating patients with absolute polycythemia. High EPO levels might be seen in both primary and secondary polycythemias. Further investigations including genetic testing are essential in distinguishing both causes of polycythemia. Our patient has an elevated EPO, which is usually seen in secondary polycythemias. Her presentation with right thalamic infarct due to a possible hyperviscosity syndrome with the lack of a clear cause of secondary polycythemia made the possibility of secondary erythrocytosis unlikely. Her BM biopsy result and JAK-2 mutation positivity confirmed the diagnosis of a myeloproliferative disorder, most likely PV.
